# Das quantifizierte EEG im elektroenzephalogrammbasierten Monitoring während Allgemeinanästhesie

**DOI:** 10.1007/s00101-021-00960-5

**Published:** 2021-05-10

**Authors:** H. A. Kaiser, J. Knapp, J. Sleigh, M. S. Avidan, F. Stüber, D. Hight

**Affiliations:** 1 Universitätsklinik für Anästhesiologie und Schmerztherapie, Inselspital, Universitätsspital Bern, Freiburgstr., 3010 Bern, Schweiz; 2 Department of Anaesthesia, Waikato Clinical School, University of Auckland, Hamilton, Neuseeland; 3 Department of Anesthesiology, Washington University School of Medicine, 660 S. Euclid Ave., MO 63110 St. Louis, USA

**Keywords:** Bewusstsein, Allgemeinanästhesie, Allgemeinanästhetika, Intraoperatives neurophysiologisches Monitoring, Dosis-Wirkung-Beziehung, Arzneimittel, Consciousness, Anesthesia, general, Anesthetics, general, Intraoperative neurophysiological monitoring, Dose-response relationship, drug

## Abstract

Das Elektroenzephalogramm (EEG) findet im klinischen Alltag der Anästhesie des deutschsprachigen Raumes zunehmend Anwendung. Bei über 90 % der Patienten ändert sich das frontale EEG als Reaktion auf die Gabe der gebräuchlichen Narkotika (Propofol und volatile Narkosegase) in typischer Weise. Eine adäquate Narkosetiefe und angemessene Konzentrationen der Anästhetika im Gehirn erzeugen meist frontale Oszillationen zwischen 8 und 12 Hz (α-Oszillationen) sowie langsame δ‑Wellen zwischen 0,5 und 4 Hz. Die frontale EEG-Ableitung eignet sich gut zur Vermeidung einer unzureichenden Narkosetiefe bzw. einer Überdosierung von Anästhetika. Im Folgenden werden die klinische Interpretation der wichtigsten EEG-Muster und ihr biophysikalischer Hintergrund erläutert. Ebenso werden wichtige Limitationen und „Fallstricke“ für den klinischen Alltag diskutiert, die der Anästhesist kennen sollte, um das EEG als zwar unvollständigen, aber klinisch äußerst wichtigen Parameter des Bewusstseinslevels zu nutzen.

## Abstract (en)

The electroencephalogram (EEG) is increasingly being used in the clinical routine of anesthesia in German-speaking countries. In over 90% of patients the frontal EEG changes somewhat predictably in response to administration of the normally used anesthetic agents (propofol and volatile gasses). An adequate depth of anesthesia and appropriate concentrations of anesthetics in the brain generate mostly frontal oscillations between 8 and 12 Hz as well as slow delta waves between 0.5 and 4 Hz. The frontal EEG channel is well-suited for avoidance of insufficient depth of anesthesia and excessive administration of anesthetics. This article explains the clinical interpretation of the most important EEG patterns and the biophysical background. Also discussed are important limitations and pitfalls for the clinical routine, which the anesthetist should know in order to utilize the EEG as an admittedly incomplete but clinically extremely important parameter for the level of consciousness.

## Lernziele

Nach Lektüre dieses CME-Beitrags werden Sie …

- die typischen Veränderungen des Elektroenzephalogramms (EEG), die mit einer Allgemeinanästhesie mit volatilen oder i.v.-Anästhetika einhergehen, kennen.
- in der Lage sein, eine zu tiefe oder zu oberflächliche „Hypnose“ während einer Allgemeinanästhesie, basierend auf dem EEG, zu erkennen.
- Informationen aus dem rohen EEG, seinem abgeleiteten Leistungsspektrum und Spektrogramm verwenden können, um die Verabreichung volatiler oder i.v.- Anästhetika zu steuern.
- auch seltenere Situationen erkennen, in denen die frontalen EEG-Merkmale anscheinend nicht mit dem klinischen Verhalten des Patienten übereinstimmen.
- die Limitierungen des frontalen EEG kennen.

## Einführung

Angesichts der Tatsache, dass das Gehirn mit das wichtigste Organ des Menschen ist, überrascht es, dass es das einzige lebenswichtige Organ ist, dessen Funktion nicht routinemäßig im OP oder auf der Intensivstation überwacht wird. Die plausibelste Erklärung ist, dass im Gegensatz zu Nieren, Herz, Lungen oder Leber bisher keine Parameter generiert werden konnten, die einen sicheren Anhalt über die pharmakologisch induzierte **Bewusstlosigkeit**

Bewusstlosigkeit

ergeben. In der klinischen Medizin können bestimmte Hirnfunktionen nur dann zuverlässig überwacht werden, wenn die Patienten bei Bewusstsein sind. Durch ihre Interaktion mit der Umwelt lässt sich allgemein recht verlässlich feststellen, wie gut ihre Hirnfunktion ist. Diese Möglichkeit der Überwachung fehlt in der Anästhesie und Intensivmedizin aber sehr häufig, und von daher sollten Kenntnisse über das Elektroenzephalogramm (EEG) als Messverfahren für die **neuronale Aktivität**

neuronale Aktivität

und des Bewusstseins für jede Anästhesistin und jeden Anästhesisten von großem Interesse sein.

Im EEG wird die summierte, synchrone **elektrische Aktivität**

elektrische Aktivität

von Millionen von Neuronen der Großhirnrinde nichtinvasiv von der Kopfhaut des Patienten aus dargestellt. Bereits 1937 empfahlen die Anästhesisten Gibbs, Gibbs und Lennox, das EEG routinemäßig zu verwenden, um die **anästhetikainduzierte „Hirnfunktionsstörung“**

anästhetikainduzierte „Hirnfunktionsstörung“

zu überwachen [1]. In den letzten 3 Jahrzehnten haben Anästhesistinnen und Anästhesisten aber erst damit begonnen, diese Zusatzinformation routinemäßig zu nutzen, um die Durchführung einer Allgemeinanästhesie zu steuern. Hierbei kam es inzwischen aber im klinischen Alltag häufig zu einer Abwendung von der klassischen EEG-Interpretation hin zur Verwendung des „vereinfachten“ *prozessierten* EEG (mit einem Index von 0: Koma bis 100: wach).

### Merke

Es wird zwischen dem klassischen und quantitativen EEG sowie dem vereinfachten prozessierten EEG unterschieden.

Da der Berechnung dieses *prozessierten* EEG komplexe Algorithmen zugrunde liegen, ergeben sich daraus häufig Probleme bei der Interpretation im klinischen Alltag. Im Rahmen dieses Weiterbildungsbetrags soll daher der aktuelle Wissensstand über die Verwendung des *klassischen* und des *quantitativen* EEG während einer Allgemeinanästhesie zusammengefasst und für den klinischen Alltag interpretierbar gemacht werden. Zuerst konzentriert sich der Beitrag auf die bekannten Veränderungen im frontalen EEG mit zunehmender Anästhetikakonzentration, während anschließend erläutert wird, wie sich das EEG mit zunehmender oder abnehmender Anästhetikakonzentration meist in typischer Weise verändert. Der letzte Abschnitt behandelt ungelöste Probleme und zukünftige Entwicklungen der EEG-Überwachung während der Allgemeinanästhesie.

### Fallbeispiel

Sie betreuen einen 58-jährigen Patienten zur laparoskopischen Hemikolektomie. Als relevante Vorerkrankungen sind Bluthochdruck und Adipositas (Body-Mass-Index 37 kg/m^2^) bekannt. Der Patient erhält für den Eingriff eine Kombinationsanästhesie aus „Transversus-abdominis-plane“(TAP)-Blockade und Allgemeinanästhesie mit Sevofluran und Fentanyl. Die Einleitung verläuft problemlos, aber während der Insufflation von Kohlendioxid in das Peritoneum wird der Patient ausgeprägt hypoton, mit einem Blutdruck von 65/35 mm Hg. Sie vermuten als Ursache primär das Kapnoperitoneum, aber ein Blick auf den EEG-Monitor führt Sie zu einer weiteren Differenzialdiagnose …

Trotz einer minimalen alveolären Konzentration (MAC) des Sevoflurans von nur 0,8 weist das EEG ein deutlich erkennbares Burst-Suppression-Muster auf. Nach einmaliger Gabe von 10 µg Noradrenalin und Reduktion der Sevoflurankonzentration auf 0,6 MAC stabilisiert sich der Blutdruck.

Während der nächsten 90 min verläuft die Anästhesie ohne größere hämodynamische Veränderungen, und das EEG zeigt stabile α‑Oszillationen und eine „Slow-wave“-Aktivität. Operativ erschweren dem Chirurgen abdominelle Adhäsionen die Arbeit. Nachdem es zu einer Verletzung des Dünndarms kommt, entscheidet sich der Operateur für ein offenes Verfahren und führt eine Laparotomie durch. Hierbei verändert sich das EEG-Muster abrupt, und es sind nur noch δ-Wellen zu erkennen. Sie sind sich unsicher, ob Sie die Narkose durch die Gabe von Fentanyl oder eine höhere Konzentration von Sevofluran anpassen sollen. Auf einen erneuten Bolus an Opioid erscheinen wieder α‑Spindeln im EEG. Anscheinend war die TAP-Blockade nicht ausreichend für die Abdeckung des gesamten Laparotomiebereiches, und der akute Schmerzreiz hat zum Verlust der α‑Oszillationen geführt.

Die Operation verläuft weiterhin gut, bis es aufgrund einer Lazeration der V. cava inferior zu einem ausgeprägten Blutverlust kommt. Aufgrund der hämodynamischen Instabilität verringern Sie die Sevoflurankonzentration auf etwa 0,4 MAC und versuchen, den Patienten durch Infusion von Kristalloiden und Transfusion von Erythrozytenkonzentrat zu stabilisieren. Ihnen fallen jedoch beim Blick auf den Monitor erneut fehlende α‑Oszillationen im EEG auf, wobei im Spektrogramm dieses Mal dem „α-Verlust“ ein Anstieg der Frequenz der α‑Oszillationen vorausging. Sie erhöhen daher die Konzentration des volatilen Anästhetikums wieder auf 0,5 MAC, trotz weiterhin bestehender hämodynamischer Instabilität, und supplementieren mit einem Bolus von 2 mg Midazolam, sodass wieder α‑Oszillationen im EEG zu erkennen sind. Nach chirurgischer Kontrolle der Blutung stabilisiert sich der Patient zusehends; nach der Ausleitung ist er schmerzfrei und kann sich nur an Ihre Worte vor der Einleitung erinnern.

## Drei zentrale Darstellungsformen des Elektroenzephalogramms

1. *Roh-EEG* (Amplitude über der Zeit, „Zeitbereich“ oder „time domain“),
2. *Leistungsspektrum* (Leistung [„power“] über die Frequenz, „Frequenzbereich“ oder „frequency domain“),
3. *Spektrogramm* (Leistung pro Frequenz über die Zeit, Leistungsdichtespektrum).

Es gibt drei klassische Arten der Darstellungsform des EEG, die in diesem Beitrag behandelt werden. Die erste besteht darin, die Schwankungen der EEG-Amplitude (gemessen in Mikrovolt, µV) im Zeitverlauf zu betrachten. Dies wird als **Roh-EEG**

**Figure 1:**
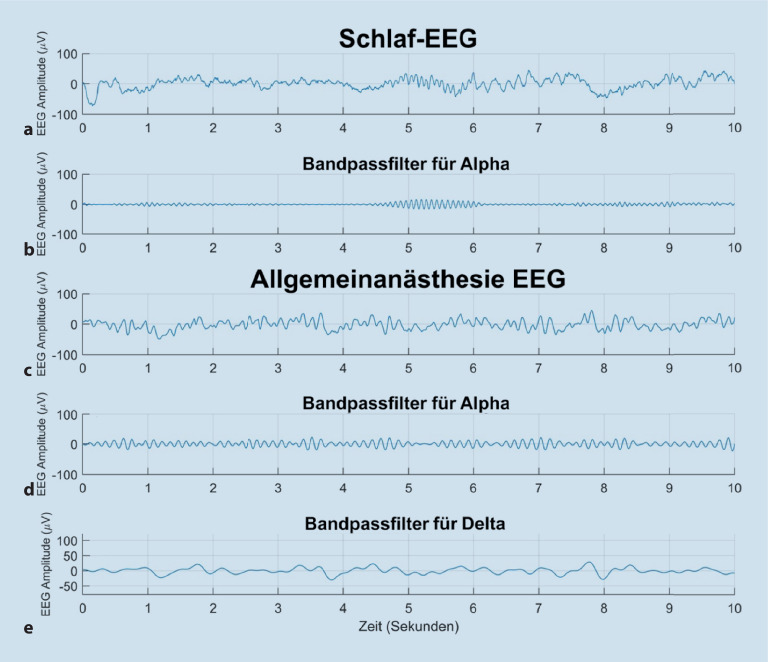

Roh-EEG

bezeichnet (Beispiele in Abb. 1, 2 und 3). In dieser Darstellung (auch als „Zeitbereich“ oder „time domain“ bezeichnet) können die EEG-Wellen-Morphologie beurteilt und die Frequenz einer Schwingung visuell abgeschätzt bzw. die genaue Zahl der Schwingungsspitzen pro Sekunde abgezählt und so die Frequenz bestimmt werden (Abb. 3).

**Figure 2:**
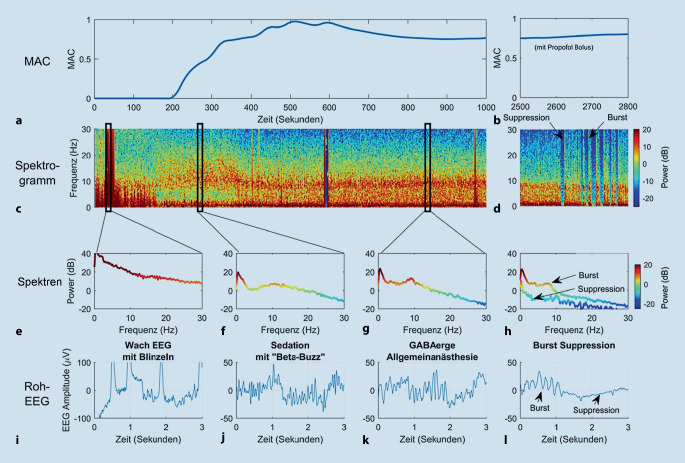

**Figure 3:**
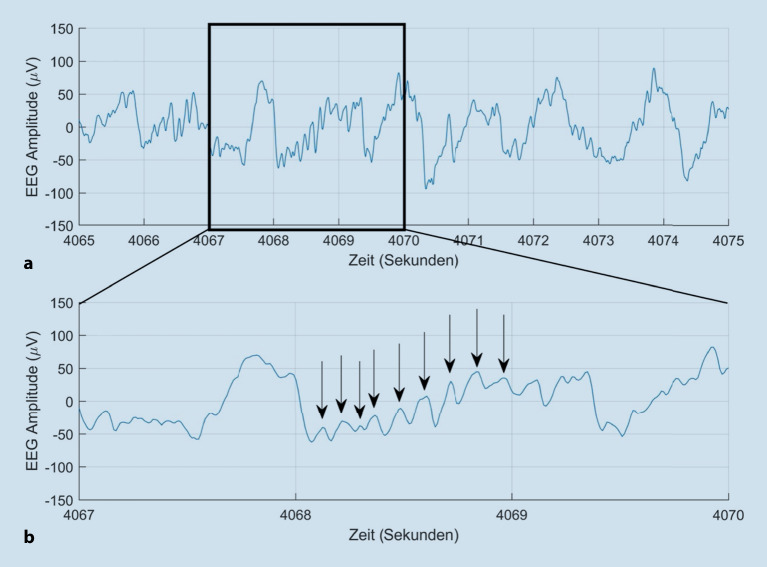

Da ein charakteristisches EEG während der Allgemeinanästhesie aus einer Kombination von Schwingungen mit unterschiedlichen Amplituden und Frequenzen besteht, ist eine zweite Ansicht hilfreich: das **Leistungsspektrum**

Leistungsspektrum.

Um ein Leistungsspektrum (auch als „Frequenzbereich“ oder „frequency domain“ bezeichnet) zu erstellen, wird ein Abschnitt des EEG (z. B. 5 s) unter Verwendung der **Fast-Fourier-Transformation**

Fast-Fourier-Transformation

(FFT) analysiert und so ein Leistungswert (in Dezibel [dB], früher meist in Quadrat der Amplitude [µV^2^]) für jeden Frequenzbereich berechnet. Da das Leistungsspektrum das genaue Ausmaß darstellt, welcher Frequenzbereich im analysierten EEG-Abschnitt mit wie viel Leistung vertreten ist, kann es manchmal Frequenzinhalte aufdecken, die im Roh-EEG möglicherweise nicht mit dem bloßen Auge erkannt werden können (Abb. 2).

Die dritte Darstellungsform des EEG – das **Leistungsdichtespektrum**

Leistungsdichtespektrum,

oder häufig vereinfacht *Spektrogramm* genannt – zeigt, wie sich die Leistung jeder Frequenz im EEG über die Zeit ändert. Um diese Darstellungsform zu erstellen, werden die Werte aus dem Leistungsspektrum farbcodiert und hohe Leistungen meist rot bzw. niedrige Leistungen blau dargestellt (Abb. 2). Hierzu werden fortlaufend EEG-Abschnitte mithilfe der FFT analysiert, um aufeinanderfolgende Leistungsspektren zu erzeugen, die zeitlich hintereinander gereiht, quasi „von oben“, betrachtet werden können, wobei die Frequenz auf der y‑Achse, die Zeit auf der x‑Achse und die Leistung als Farbskala als dritte Ebene dargestellt werden. Der Vorteil dieser Perspektive besteht darin, dass sie über lange Zeiträume (d. h. Stunden) geringfügige Änderungen der Frequenz- und Leistungswerte des EEG aufzeigen kann.

### Merke

Drei Darstellungsformen des EEG sind für die Anästhesie relevant: das Roh-EEG, das Leistungsspektrum (oft nur „Spektrum“ abgekürzt) und das Spektrogramm.

## Veränderungen im frontalen EEG während einer Allgemeinanästhesie

### Klassische Schwingungsmuster bei steigender Anästhetikakonzentration

Eine gängige Nomenklatur zur Beschreibung der **Frequenzbänder**

Frequenzbänder

oder Komponenten, die in komplexen EEG-Wellen vorkommen, ist in Tab. 1 aufgeführt. Diese Frequenzbänder sind willkürlich definiert und haben keine spezifische neurobiologische Bedeutung.

**Table 1:** 

Name des Frequenzbands	Frequenzband in Hertz
„Slow waves“	0–1 Hz
Delta (δ)	1–4 Hz (bisweilen auch 0–4 Hz definiert)
Theta (θ)	4–8 Hz
Alpha (α)	8–12 Hz
Sigma (σ)	12–14 Hz (oft das Frequenzband für Schlafspindeln während des physiologischen Schlafs)
Beta (β)	Im Allgemeinen 12–30 Hz
Gamma (γ)	> 30 Hz (bis > 100 Hz)

Für die Anästhesie sind v. a. folgende 3 Schwingungs- bzw. Oszillationsmuster relevant:

- langsame Wellen, „slow waves“ (< 1 Hz),
- δ*-Wellen* (1–4 Hz) und
- kontinuierliche Spindeln im α‑Frequenz-Bereich („α-Spindeln“, meist zwischen 8 und 12 Hz).

Bei Verwendung des Begriffs **„α-Spindel“**

„α-Spindel“

gibt es 2 wichtige Punkte zu beachten, um Missverständnisse zu vermeiden:

1. Die „α-Spindeln“ bzw. Oszillationen im α‑Frequenz-Bereich während einer Allgemeinanästhesie können manchmal langsamer oder schneller sein als 8 bzw. 12 Hz (z. B. sie verlangsamen sich mit zunehmendem Alter und v. a. mit zunehmender Konzentration an Anästhetika).
2. Die durch GABAerge Anästhetika induzierten „α-Spindeln“ unterscheiden sich von klassischen Schlafspindel des Schlafstadiums N2 dahingehend, dass sie kontinuierlicher (Abb. 1d) sowie nur frontal und nicht, wie in Abb. 1b gezeigt, nur punktuell auftreten (Sekunde 4,5–6 des Schlaf-EEG).

#### Cave

α-Spindeln können mit zunehmendem Patientenalter und Anästhestika-Konzentration niedrigerfrequent werden als 8 Hz.

Zur Veranschaulichung dieser 3 für die Anästhesie relevanten Schwingungsmuster ist in Abb. 3 ein exemplarisches Roh-EEG dargestellt. Die Kurve in Abb. 3a zeigt 10 s eines Roh-EEG, die Kurve in Abb. 3b einen Ausschnitt von 3 s hieraus in höherer Auflösung. In Abb. 3a ist die langsame „Grundschwingung“ der **Slow wave**

Slow wave

zu erkennen. Die etwa jede Sekunde einmal auftretende Amplitude von ca. 100 µV entspricht der δ‑Aktivität (ca. 1 Hz). In der Kurve der Abb. 3b ist durch die Vergrößerung die α‑Aktivität besser zu erkennen (mit *Pfeilen* markiert). Sie ist im Vergleich zur δ‑Aktivität durch kleinere Spannungsschwankungen (um 40 µV) gekennzeichnet, die in diesem Ausschnitt 9‑mal/s auftreten (9 Hz).

Bei steigenden Dosen von GABAergen (auf γ‑Aminobuttersäure [GABA] reagierende Rezeptoren wirkende) Anästhetika wie den volatilen Anästhetika auf Ätherbasis oder Propofol [3, 4], zeigt die überwiegende Mehrheit der Patienten typische Veränderungen im frontalen EEG. Daher eignet sich das EEG zur Titration der korrekten **Anästhetikadosierung**

Anästhetikadosierung.

Dies kann sowohl einer unzureichenden als auch übermäßigen Verabreichung von Anästhetika mit ihren potenziellen klinischen Folgen (z. B. Kreislaufdepression, Awareness, postoperative kognitive Dysfunktion) vorbeugen.

#### Merke

Das EEG eignet sich gut zur korrekten Titration von Anästhetika.

Die GABAergen Anästhetika bewirken eine Hyperpolarisation von thalamischen und kortikalen Neuronen, wodurch die Weiterleitung des chirurgischen **Schmerzreizes**

Schmerzreizes

vom Thalamus zum Kortex eingedämmt wird. Im EEG spiegelt sich diese Hyperpolarisation durch die Erzeugung von α‑ und δ‑Wellen wider [5]. Die derzeitige Hypothese ist, dass α*-Spindeln* aus sich wiederholenden abwechselnden Spike- und Burst-Mustern aus retikulären Thalamusneuronen resultieren, die rhythmische inhibitorische postsynaptische Potenziale erzeugen [6]. Die zusätzlichen **δ‑Oszillationen**

δ‑Oszillationen,

die ebenfalls während der Anästhesie im EEG beobachtet werden (Abb. 1e), könnten ein stärker hyperpolarisiertes Niveau der Membranpotenziale der thalamokortikalen Neurone anzeigen, als wenn nur α‑Spindeln erzeugt werden [7].

Bei niedrigen Anästhetikakonzentrationen, die nur eine leichte Sedierung bewirken, wird die frontale EEG-Wellenform typischerweise zu Beginn von einer langsamen **β-Aktivität**

β‑Aktivität

dominiert, die eine Frequenz um 12–20 Hz aufweist. Der Patient kann während dieser Phase Anzeichen einer **paradoxen Erregung**

paradoxen Erregung

zeigen. Wenn der Patient mit steigenden Zielortkonzentrationen an Reaktionsfähigkeit verliert, ist frontal normalerweise eine Verlangsamung der β‑Oszillationen hin zu **α-Oszillationen**

α‑Oszillationen

(8–12 Hz) zu beobachten (Abb. 2j, k; [8, 9]). Wenn dahingegen eine Person mit geschlossenen Augen wach und entspannt ist, sind im EEG typischerweise posterior dominante α‑Oszillationen über okzipitalen und parietalen Regionen zu erkennen. Während der Einleitung einer Allgemeinanästhesie verschiebt sich zum Zeitpunkt des Reaktionsverlustes diese dominante Oszillation im α‑Frequenz-Band von okzipitalen und parietalen Regionen zu frontalen Regionen des Gehirns. Dieses Phänomen ist als **„α-Anteriorisierung“**

„α-Anteriorisierung“

bekannt [10] und macht eine aussagekräftige Überwachung mithilfe des frontalen EEG während der Anästhesie erst möglich.

Nach dem Verlust der Reaktionsfähigkeit treten im EEG ebenfalls langsamere Schwingung im Slow-waves- (0–1 Hz) und im δ‑Bereich (1–4 Hz) auf (Abb. 2k und 3). Eine weitere Erhöhung der hypnotischen Dosis führt zu Phasen der EEG-Suppression (d. h. äußerst niedrige Amplitude, meist weniger als 5 µV), die von „bursts“ mit hoher Amplitude (> 20 µV) unterbrochen werden. Dementsprechend nennt man dieses Muster auch **Burst-Suppression**

Burst-Suppression

(Abb. 2d, h, l). Eine weitere Dosiserhöhung resultiert letztendlich in einem kontinuierlich unterdrückten oder **isoelektrischen EEG**

isoelektrischen EEG

(völliges Fehlen sichtbarer Schwingungen). Im Leistungsspektrum (Abb. 2h) erscheinen Bursts als Perioden hoher Leistung über alle Frequenzen und Bereiche der Suppression als Perioden niedriger Leistung über alle Frequenzen. Im Spektrogramm (Abb. 2d) werden die Suppressionsperioden als scharf definierte senkrechte Streifen kälterer Farben (*blau*) angezeigt.

Diese klassischen EEG-Reaktionen auf aufsteigende Anästhetikakonzentrationen wurden im Laufe der Jahrzehnte in vielen Publikationen detailliert beschrieben. Zu den bemerkenswertesten und umfassendsten frühen Arbeiten zählen die von Rampil [11] sowie Jameson und Sloan [12]. Brown et al. [13] konzentrierten sich in den vergangenen Jahren v. a. auf Bahnen des aktivierenden Systems, die an der Erzeugung verschiedener EEG-Muster beteiligt sind, während Übersichten mit einer klinischeren Sichtweise die von Bennett et al. [14], Jagadeesan et al. [15], Marchant et al. [16] und Purdon et al. [17] sind. Diese beschreiben die Veränderungen im rohen EEG-Signal [14] als auch die Veränderungen der Zusammensetzung der Frequenzen im EEG-Signal über die Zeit (v. a. im Spektrogramm [17]). Außerdem werden in diesen Berichten verschiedene Arten an Artefakten beschrieben, die sich im EEG manifestieren können (z. B. Blinzeln [Abb. 2i]) und auch, wie sich das EEG als Reaktion auf nicht-GABAerge Anästhetika wie Lachgas und Ketamin verändert. Auf den Websites http://icetap.org und www.anesthesiaEEG.com finden sich ebenfalls nützliche weiterführende Informationen zum EEG während einer Anästhesie.

### Unzureichende Dosierung der Anästhetika

Die Überwachung der endtidalen Konzentration von Inhalationsanästhetika und die Aufrechterhaltung angemessener an das Patientenalter angepasster Konzentrationen sind zentrale Aufgaben jeder Anästhesistin bzw. jedes Anästhesisten, um Fälle mit expliziter Erinnerung – **Awareness**

Awareness

 – an ein intraoperatives Ereignis während Allgemeinanästhesie zu vermeiden. Ein besonderes Risiko für eine Unterdosierung besteht bei totaler intravenöser Anästhesie (TIVA) in Kombination mit neuromuskulärer Blockade und wenn die endtidale Konzentration volatiler Anästhetika nicht adäquat gemessen werden kann, so z. B. während der Übergangsphase von der Herz-Lungen-Maschine zur maschinellen Beatmung (einer **„Mind-the-gap“-Situation**

**Figure 4:**
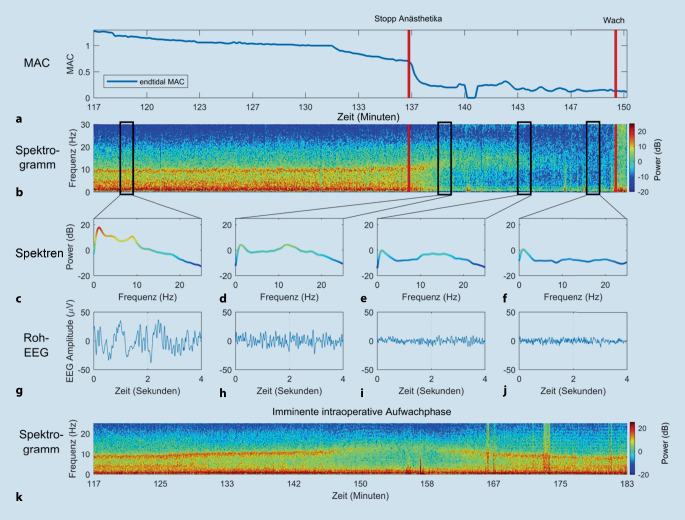

„Mind-the-gap“-Situation,

Abb. 4k; [18, 19]) oder wenn – wie im Fallbeispiel – die Anästhetikakonzentration aus hämodynamischen Gründen reduziert wird.

#### Cave

Bei TIVA in Komination mit Muskelrelaxanzien, bei Eingriffen mit extrakorporaler Zirkulation und in hämodynamisch instabilen Situationen besteht besondere Gefahr von Awareness.

Wenn die Dosis des verabreichten Anästhetikums stetig verringert wird, nehmen die Frequenz der α‑Spindeln zu und die Amplitude ab, bevor sie intermittierend und zuletzt ganz verschwinden (Abb. 4g–j). Die Veränderung der α‑Spindel-Frequenz in Abhängigkeit von der Zielortkonzentration ist mit zunehmendem **Alter**

Alter

und **Komorbiditäten**

Komorbiditäten

(z. B. Diabetes mellitus, chronische Niereninsuffizienz, zerebrovaskuläre Verschlusskrankheit oder pulmonalarterieller Hypertonus) aufgrund der damit verbundenen verminderten Leistung im EEG oft schwieriger zu erkennen als bei jungen Patienten ohne die genannten Begleiterkrankungen [20, 21, 22].

### Übermäßige Dosierung von Anästhetika

Die Verwendung des intraoperativen EEG kann dazu beitragen, eine mögliche Überdosierung des Anästhetikums v. a. bei sensitiven, d. h. meist älteren und komorbiden, Patienten zu vermeiden. Obwohl retrospektiv ein Zusammenhang zwischen der Dauer der intraoperativen EEG-Suppression und der Inzidenz des postoperativen Deliriums beobachtet wurde [23] ist noch unklar, ob eine längerfristige EEG-Suppression schädlich ist [24].

Das offensichtlichste, wenn auch unspezifische EEG-Merkmal einer übermäßigen Dosierung von Anästhetika ist die sog. Burst-Suppression oder die anhaltende Suppression mit isoelektrischem EEG. Da das ursprüngliche Maß der **minimalen alveolären Konzentration**

minimalen alveolären Konzentration

(MAC) auf der Grundlage der fehlenden Bewegung bzw. Reaktion auf einen Schmerzreiz abgeleitet wurde, basiert die Dosierung somit zu einem gewissen Grad auf der Wirkung der volatilen Anästhetika auf das **Rückenmark**

Rückenmark

und nicht unbedingt auf den Kortex [25]. Aus diesem Grund könnte die Einführung eines MAC-Maßes, das auch den Grad an EEG-Suppression miteinberechnet, ein zuverlässigerer Parameter für den tatsächlichen Wirkorteffekt volatiler Anästhetika sein [26]. Das EEG-Monitoring kann der Anästhesistin/dem Anästhesisten einen Anhalt dafür geben, inwieweit die **Kortexaktivität**

Kortexaktivität

eines bestimmten Patienten als Reaktion auf eine bestimmte Anästhetikakonzentration unterdrückt wird. Somit können die Patienten identifiziert werden, die aufgrund systemischer Erkrankungen oder anderen Gründen unerwartet empfindlich auf Anästhetika reagieren.

#### Merke

Das EEG gibt einen Anhalt über die Suppression der Kortexaktivität und ermöglicht so die Identifikation von besonders Anästhetika-empfindlichen Patienten.

### Bekannte Artefakte

Das EEG-Signal reflektiert nicht nur die elektrische Aktivität, die vom Gehirn ausgeht, sondern es kann natürlich auch elektrische Aktivität aus anderen Quellen als dem Gehirn enthalten, z. B. Herz- und Skelettmuskulatur, Konvektionswärmer, Bewegungen oder Berührungen.

So genannte Fremdgeräusche entstehen durch alle Arten von **elektrischen Geräten**

elektrischen Geräten

im chirurgischen Umfeld; z. B. aus der Elektrokauterisation im Operationsfeld (ersichtlich als Breitbandrauschen über alle Frequenzbänder) und aus dem Elektrokardiogramm. Artefakte werden auch durch **Bewegungen**

**Figure 5:**
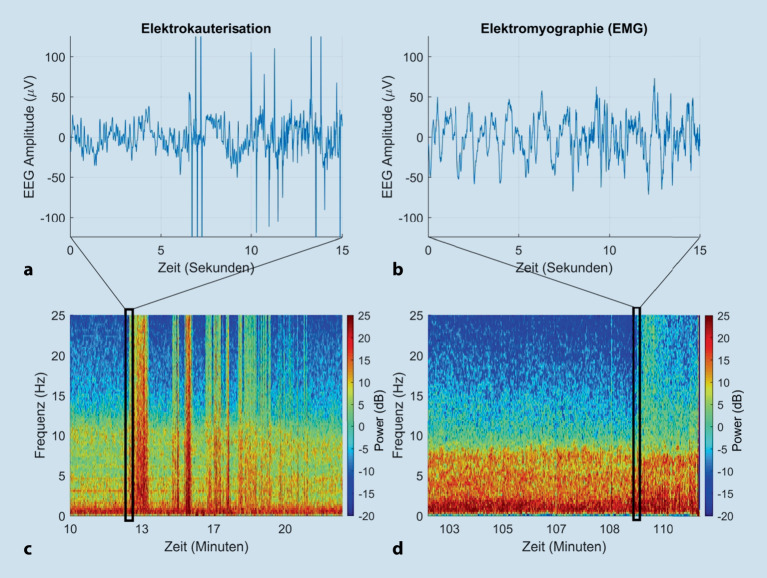

Bewegungen

des Patienten und Berührung der EEG-Elektroden induziert. Dies führt zu ausgeprägten, sehr sprunghaften Anstiegen der Amplitude im EEG, die eine Interpretation vorübergehend verunmöglichen. Beispiele, wie die Elektrokauterisation sowohl im Roh-EEG als auch im Spektrogramm aussieht, zeigt Abb. 5.

Muskelaktivität wird intraoperativ elektrisch als **Elektromyographiesignal**

Elektromyographiesignal

(EMG-Signal) erfasst. Das EMG-Monitoring kann das EEG-Signal „unscharf“ erscheinen lassen, da dieses Artefakt meist im Hochfrequenzbereich (> 20 Hz) liegt. Im Spektrogramm zeigt sich die Muskelaktivität als erhöhte Leistung über mehrere Frequenzbänder („Breitbandrauschen“, Abb. 5d). Sowohl Anästhetika als auch **neuromuskuläre Blocker**

neuromuskuläre Blocker

können die Muskelaktivität und damit das Ausmaß der EMG-Kontamination des EEG-Signals reduzieren.

Ferner gibt es vereinzelt Patienten, die von Beginn an ein EEG mit kleiner Amplitude vorweisen. Bekannt ist, dass die EEG-Amplitude bzw. Leistung mit dem Alter abnehmen kann. Die verbleibende Varianz der Amplitude kann möglicherweise auf **Neurodegeneration**

Neurodegeneration

oder genetische Faktoren zurückzuführen sein. Bei solchen Patienten wird rasch fälschlicherweise der Schluss gezogen, dass das EEG supprimiert bzw. isoelektrisch sein könnte. Wird aber das EEG-Signal genauer betrachtet, während die Anästhetikakonzentration verändert wird, lässt sich manchmal ermitteln, ob es sich wirklich um Suppression oder nur niedrige Amplituden handelt. Es kann in einem solchen Fall auch sehr hilfreich sein, die Amplitudenskala (y-Achse) der EEG-Wellenform zu verringern. Eine typische Standardamplitudenskala beträgt je nach Monitor ungefähr 50–100 µV/cm auf dem Bildschirm. Das Reduzieren der Skala auf 20 µV/cm kann helfen festzustellen, ob α‑Spindeln mit kleinerer Amplitude und Slow-wave-Oszillationen vorhanden sind.

#### Cave

Einzelne Patienten (v.a. im hohen Alter oder bei neurodegenerativen Erkrankungen) zeigen im EEG eine niedrige Amplitude. Dies darf nicht mit einer Suppression verwechselt werden.

### Nicht-GABAerge Anästhetika

Bei Verwendung von Anästhetika, deren Wirkung nicht primär über den GABA-Rezeptor vermittelt wird (z. B. **Lachgas** und **Ketamin**

**Figure 6:**
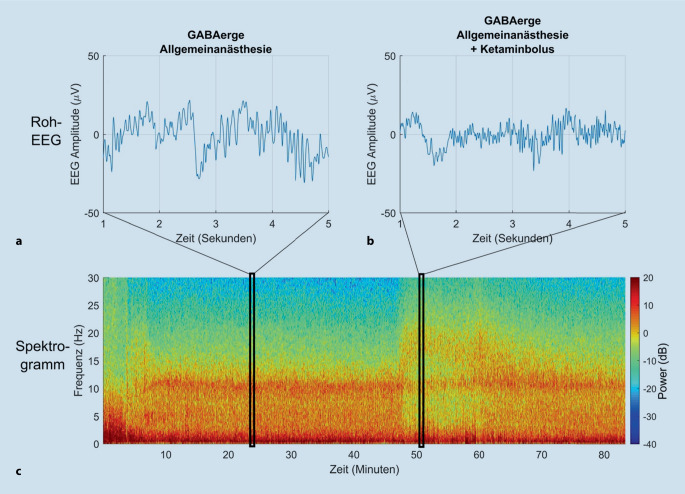

Ketamin),

zeigt das frontale EEG nicht die oben beschriebenen klassischen Merkmale, sondern es werden höhere Frequenzkomponenten mit weit weniger Leistung im α‑Bereich induziert. Die zusätzliche Gabe von Ketamin während einer Allgemeinanästhesie mit Propofol oder volatilen Anästhetika kann – je nach Dosis und Patient – eine EEG-Aktivität v. a. im β‑Bereich provozieren. Diese tritt dann typischerweise zusätzlich „im Hintergrund“ zum α‑/δ-Muster auf (Abb. 6). In solchen Fällen stellt ein EEG mit zusätzlicher Leistung im höher frequenten Bereich keinen leichteren hypnotischen Zustand des Patienten dar (an den das Spektrogramm auf den ersten Blick erinnern kann), sondern in Kombination mit GABAergen Medikamenten sogar eher einen tieferen.

#### Merke

Die zusätzliche Gabe von Ketamin während Allgemeinanästhesie provoziert eine EEG-Aktivität im β-Bereich. Dies zeigt keinen oberflächlicheren, sondern eher einen tieferen hynotischen Zustand an.

Dexmedetomidin und andere **α**_**2**_**-Agonisten**

α_2_-Agonisten

wie Clonidin erzeugen im Gegensatz dazu ein vorwiegend δ‑dominantes EEG mit intermittierenden α‑Spindeln. Das EEG während der alleinigen Gabe von α_2_-Agonisten offenbart Merkmale, die identisch sind zum **Non-REM-Schlaf**

Non-REM-Schlaf

des Stadiums N2 und N3 [17]. In Übereinstimmung mit dieser Beobachtung ist der durch **Dexmedetomidin**

Dexmedetomidin

erzeugte sedierte Zustand in gewisser Hinsicht dem des Schlafs viel näher als der Allgemeinanästhesie. Während bei höheren Konzentrationen an Dexmedetomidin die EEG-Merkmale den Eindruck einer tiefen Anästhesie (d. h. langsame Oszillationen mit großer Amplitude) erwecken können, erwacht ein Patient aber als Reaktion auf eine chirurgische Stimulation abrupt aus diesem Zustand. Wird Dexmedetomidin zusätzlich zu GABAergen Anästhetika verabreicht, ist im α‑/δ-Muster v. a. eine Verstärkung der δ‑Wellen zu beobachten.

### Unerwartete nichtlineare EEG-Reaktion auf intraoperativen Schmerzreiz

Bei adäquaten Konzentrationen an Anästhetika führt ein Schmerzreiz während einer Operation zu keiner Veränderung des EEG, falls das **analgetische Niveau**

analgetische Niveau

für den Patienten und das Ausmaß des chirurgischen Schmerzreizes ausreichend ist. Bei unzureichender Analgesie zeigt das EEG dagegen einen Verlust im α‑ und δ‑Bereich und einen Anstieg hochfrequenter Wellen (β- und γ‑Bereich). Der Anstieg der hohen Frequenzen wird als „β-Erregung“ oder **„β-Arousal“**

„β-Arousal“

bezeichnet und ähnelt dem EEG-Muster, das bei Ausleitung einer Narkose beobachtet werden kann (Abb. 4). Paradoxerweise kann es bei unzureichender Analgesie aber auch zu einer Zunahme der δ‑Aktivität kommen („δ-Erregung“ oder „paradoxe Erregung“), die sich als unerwarteter und abrupter Anstieg der δ‑EEG-Amplitude äußert [27, 28]. In dieser Situation kann der von einem EEG-Monitor berechnete Index fälschlicherweise sinken und der abrupte Verlust der α‑Spindeln mit der Situation beim Übergang zum Burst-Suppression-Muster verwechselt werden [29, 44]. Dies darf den Anästhesisten aber nicht dazu verleiten, die Konzentration der Anästhetika zu reduzieren, vielmehr sollte die analgetische Komponente der Narkose mit **Opioiden**

Opioiden

vertieft werden. Als Hypothese zur Erklärung dieses EEG-Musters wird die δ‑Erregung als ein Zeichen endogener antinozizeptiver Aktivierung diskutiert. Ein beispielhaftes Bild für eine solche Reaktion eines **„α-drop-out“**

**Figure 7:**
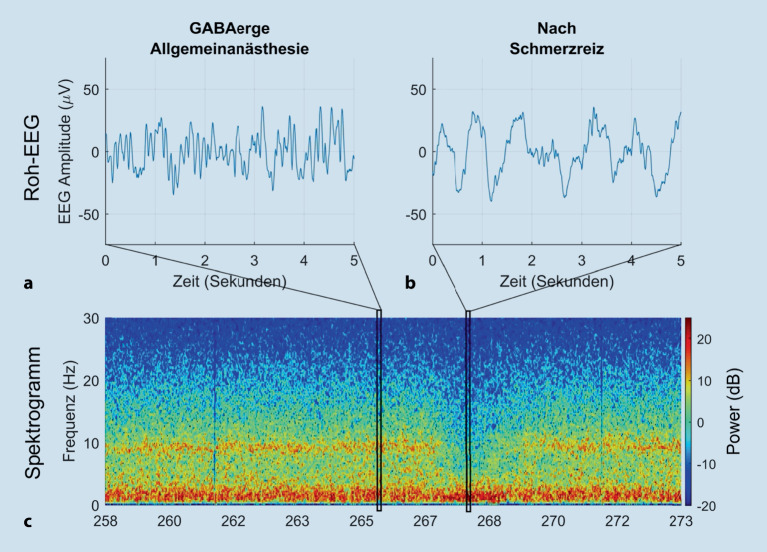

„α-drop-out“

und einer „paradoxen δ‑Erregung“ zeigt Abb. 7. Dieses Phänomen wurde bisher v. a. bei **viszeralchirurgischen Eingriffen**

viszeralchirurgischen Eingriffen

beschrieben [30, 31, 32].

#### Cave

Ein plötzlicher Verlust der α‑Spindeln und eine Erhöhung der δ-Leistung können mit dem Burst-Suppression-Muster verwechselt werden und zu einem falsch-niedrig berechneten Index im prozessierten EEG führen. Tatsächlich muss die Analgesie vertieft werden.

### Neuronale Trägheit

Während die Induktion einer Allgemeinanästhesie mit Bewusstseinsverlust eine „aktive“ medikamenteninduzierte Veränderung der Hirnfunktion darstellt, wird die Rückkehr des Bewusstseins nach einer Anästhesie oft noch als spiegelbildlicher „passiver“ Prozess wahrgenommen. Wenn diese Hypothese zutreffen würde, wären die Anästhesiekonzentrationen im Gehirn bei Bewusstseinsverlust während der Narkoseinduktion und beim Erwachen aus der Narkose identisch. Die klinische Erfahrung ebenso wie Studien an Mäusen, Fruchtfliegen [33] und Menschen [34, 35] zeigen jedoch, dass bei Wiedererlangung des Bewusstseins wesentlich niedrigere Konzentrationen an hypnotischen Anästhetika vorliegen als beim Bewusstseinsverlust. Oder anders ausgedrückt, die **MAC**_**awake**_

MAC_awake_

(„awake“: Erwachen) liegt vermutlich bei einer niedrigeren Zielortkonzentration als die MAC_LOC_ (LOC: „loss of consciousness“/Bewusstseinsverlust). Vermutlich ist das nicht nur auf die Pharmakokinetik zurückzuführen, sondern legt vielmehr nahe, dass unterschiedliche Mechanismen an den Prozessen des Bewusstseinsverlusts und des Bewusstseinswiedererlangens beteiligt sind [36]. Diese **Hysterese**

**Figure 8:**
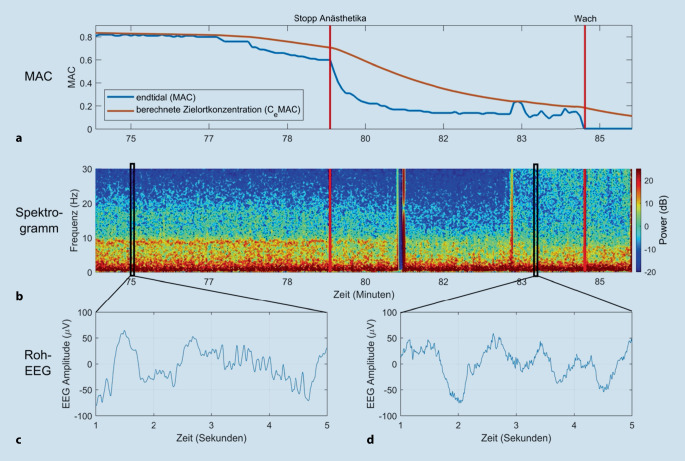

Hysterese

ist in Situationen stärker ausgeprägt, in denen aktivierende Neuromodulatoren beeinträchtigt sind (z. B. im Alter, bei Narkolepsie oder unter Droperidol, Clonidin und antimuskarinergen Medikamenten). Daher kann während der Narkoseausleitung (oder wenn die Anästhesistin oder der Anästhesist absichtlich versuchen, eine „oberflächliche“ Allgemeinanästhesie knapp unter der Schwelle des Erwachens aufrechtzuerhalten) nicht genau vorhergesagt werden, wann ein Patient zu Bewusstsein kommt. Am Beispiel einer Narkoseausleitung veranschaulicht Abb. 8 diese neuronale Trägheit.

## Reaktionsfähigkeit trotz des frontalen EEG-Musters einer adäquat „tiefen“ Anästhesie

Jüngste Untersuchungen unter Verwendung der „isolated forearm technique“ (IFT) haben gezeigt, dass vereinzelte Patienten trotz eines frontalen EEG-Musters, das eine adäquate Konzentration an Inhalationsanästhetikum anzeigt (d. h. langsame δ‑ sowie α‑Spindel-Aktivität), zu einem gewissen Grad noch bei Bewusstsein sein können, der zum Ausführen eines Befehls reicht (in diesem Fall Händedruck, [37]). Während solche Episoden recht selten sind und die klinische Relevanz solcher Zustände immer noch diskutiert wird [38], deutet dieses Phänomen aber darauf hin, dass volatile Anästhetika sogar viel eher einen **dissoziierten Zustand**

dissoziierten Zustand

verursachen können, als man bisher angenommen hat. „Philosophisch“ ausgedrückt, kann es sein, dass Anästhesistinnen und Anästhesisten nicht notwendigerweise immer die Bewusstseinsebene verringern, sondern viel mehr den **Bewusstseinsinhalt**

Bewusstseinsinhalt

modulieren [39, 40].

Eine Erklärung für diese Beobachtung könnte sein, dass δ‑ und α‑Spindel-Aktivität zwar wichtige Surrogatmarker für ein angemessenes Anästhesieniveau sind, jedoch mit dem in der Anästhesie üblichen EEG nur der frontale Bereich des Gehirns hinsichtlich des Bewusstseinsverlusts überwacht wird. Die Vorderseite der Stirn ist frei von Haaren und bietet somit einen einfachen und verlässlichen Zugang für EEG-Elektroden. Diese pragmatische Lösung bedeutet jedoch nicht automatisch den besten Ort für die Beurteilung der Bewusstlosigkeit. Einige Autoren proklamieren inzwischen eine **posteriore „hot zone“**

posteriore „hot zone“,

in der die für das Phänomen Bewusstsein verantwortlichen Neurone lokalisiert sein sollen [41]. Aber das letzte Wort zu dieser komplexen Thematik ist sicherlich noch lange nicht gesprochen.

Eine weitere Möglichkeit könnte sein, dass Bewusstlosigkeit nicht nur mit einem spezifischen EEG-Muster an einem einzelnen Ort vergesellschaftet ist, sondern eher von der **Konnektivität**

Konnektivität

zwischen 2 oder mehr Hirnregionen abhängt. Beispielsweise wurde beobachtet, dass EEG-Messgrößen für die Konnektivität zwischen frontalen und parietalen Regionen abnehmen, wenn ein Verlust der Kontaktierbarkeit eines Patienten auftritt, und dies nicht nur bei der Verwendung von Sevofluran und Propofol, sondern auch beim nicht-GABAergen Ketamin [42]. Offensichtlich wurde die genauen neuronalen Korrelate der Bewusstlosigkeit noch nicht gefunden. Diese Vorsichtsmaßnahmen und ungelöste Probleme dürfen aber kein Grund dafür sein, das EEG bei Patienten mit einer klassischen Dosis-Wirkung-Beziehung zu ignorieren [43].

## Fazit für die Praxis

- Ansätze, die in der Vergangenheit verfolgt wurden, wie z. B. die Fourier-Analyse zur Zerlegung der komplexen EEG-Wellenform in willkürlich definierte Frequenzbänder, können mit dem Verlust wertvoller Informationen verbunden sein. Es gibt höchstwahrscheinlich keinen einheitlichen Mechanismus der Anästhesiewirkung für die verschiedenen Anästhetika und deren Kombinationen. Ebenfalls ist äußerst unwahrscheinlich, dass es eine einzige EEG-Signatur gibt, die für alle Patienten jedes Alters gilt und unabhängig von Arzneimittelkombinationen ist. Für Anästhesistinnen und Anästhesisten ist es daher wichtig, sowohl die Stärken des EEG als auch seine Grenzen zu kennen.
- Es überrascht nicht sonderlich, dass das komplizierteste Organ des Körpers relativ schwierig während einer Allgemeinanästhesie überwacht werden kann. Auch ist es kaum gerechtfertigt zu behaupten, dass ein berechneter EEG-Index zwischen 0 und 100 ausreicht, um die EEG-Variabilität von Patienten, Eingriffen und Medikamenten widerzuspiegeln.
- Inzwischen hat sich ein differenzierteres Wissen bezüglich der Brauchbarkeit des „rohen“ EEG als Monitor-Verfahren einer personalisierten Allgemeinanästhesie einschließlich der potenziellen Limitierungen aktueller EEG-Anwendungen entwickelt.
- Wenn Anästhesistinnen und Anästhesisten danach streben, ihre Patienten im OP oder auf der Intensivstation mit personalisierter Medizin zu versorgen, müssen ein individuelles EEG, Leistungsspektrum und Spektrogramm einbezogen werden.

## CME-Fragebogen

Zu Beginn einer laparoskopischen Cholezystektomie weist der Patient das in Abb. 9 ersichtliche frontale EEG-Muster bei 0,9 MAC auf. Der Blutdruck beträgt 75/40 mm Hg. Wie sind die Befunde in dieser Situation zu deuten?

Die begleitende Hypotonie ist sicherlich auf die Anlage des Pneumoperitoneums zurückzuführen.

Das Belassen der 0,9 MAC führt sehr wahrscheinlich zu einem postoperativen Delir.

Das rohe EEG zeigt ein Artefakt aufgrund des Pneumoperitoneums.

Das sog. Burst-Suppression-Muster ist mit einer übermäßigen Verabreichung des volatilen Anästhetikums vereinbar.

Die EEG-Signatur ist in diesem Fall ein Zeichen für eine akute Hirnschädigung.

Während einer Allgemeinanästhesie zeigt ein Patient vornehmlich δ‑ und α‑Wellen wie im abgebildeten Spektrogramm, Spektrum und EEG ersichtlich (Abb. 10). Die endtidale Sevoflurankonzentration ist auf 0,6 MAC eingestellt. Wie sind die Befunde in dieser Situation zu deuten, und wie gehen Sie weiter vor?

Die Konzentration des volatilen Anästhetikums sollte weiterreduziert werden, bis die α‑Oszillationen verschwinden und δ‑Wellen überwiegen.

Es sollte versucht werden, den Patienten bis zum Ende der Operation in diesem EEG-Muster zu halten.

Ein schmerzhafter Reiz wird keinen Einfluss auf dieses EEG-Muster haben.

Die frontalen α‑Spindeln deuten auf ein hohes Risiko für eine intraoperative Awareness hin.

Eine Reduktion der Konzentration an volatilen Anästhetika verlangsamt tendenziell die Frequenz der α‑Spindel.

Während einer laparoskopischen Operation erfolgt aufgrund von Komplikationen die operative Konversion zur Laparotomie. Währenddessen zeigt der Patient plötzlich das in Abb. 11 dargestellte folgende Roh-EEG und Spektrogramm. Wie sind die Befunde in dieser Situation zu deuten?

Es handelt sich um eine mangelnde Dosierung des volatilen Anästhetikums; der Patient scheint kurz vor dem Erwachen zu sein.

Es könnte sich um Anzeichen einer irreversiblen Hirnschädigung handeln.

Der Patient weist ein Burst-Suppression-Muster auf, weshalb die α‑Oszillationen im rohen EEG und im Spektrogramm nicht mehr sichtbar sind.

Dieses EEG ist charakteristisch für toxische Konzentrationen an Lokalanästhetika.

Die Analgesie ist anscheinend nicht ausreichend, um den Schmerzreiz der Laparotomie abzudecken.

Im Verlauf einer viszeralchirurgischen Operation kommt es aufgrund einer Blutungskomplikation zu einer hämodynamischen Instabilität des Patienten. Im Spektrogramm (Abb. 12) zeigen sich EEG-Veränderungen. Wie ist dieser Befund in dieser Situation zu deuten?

Die dargestellten Veränderungen im EEG sind auf eine Hypoperfusion des Gehirns zurückzuführen.

Der Patient ist hypotherm, und dies ist im EEG und im Spektrogramm zu sehen.

Die Erhöhung der α‑Spindel-Frequenz ist ein typisches Muster für eine Erhöhung der Sensibilität des Gehirns auf das Anästhetikum.

Es gibt ein starkes δ‑Wellen-Muster im EEG, sodass das hypnotische Level definitiv ausreichend ist.

Während eines solchen Zeitraums besteht eine höhere Wahrscheinlichkeit für intraoperative Awareness.

Am Ende einer Operation mit Allgemeinanästhesie drehen Sie den Verdampfer zu und erhöhen die Flussrate an Frischluft am Beatmungsgerät. Obwohl die endtidale Anästhetikakonzentration rasch von 0,5 auf 0,2 MAC sinkt, zeigt das Roh-EEG, wie in Abb. 13 ersichtlich, weiterhin δ‑Wellen mit hoher Amplitude. Wie ist dieser Befund in dieser Situation zu deuten?

Der Patient ist noch weit davon entfernt, ansprechbar zu werden, da eine starke δ‑Aktivität ein Zeichen für eine tiefe Anästhesie ist.

Eine Abnahme der δ‑Leistung ist im EEG immer vor der Rückkehr der Reaktionsfähigkeit zu sehen.

δ‑Oszillationen allein sind ein gutes Maß für eine angemessene Anästhesietiefe.

Die endtidale Anästhetikakonzentration ist bei Rückkehr des Bewusstseins niedriger als beim Bewusstseinsverlust während der Einleitung.

Die dargestellten EEG-Veränderungen weisen auf eine Hypoperfusion des Gehirns hin.

Das Phänomen der Anteriorisierung wurde bereits 1977 beschrieben. Worum handelt es sich hierbei?

Während der Einleitung einer Allgemeinanästhesie verschieben sich die dominanten δ‑Wellen von okzipital/parietal zu den frontalen Regionen des Gehirns.

Während der Einleitung einer Allgemeinanästhesie verschieben sich die dominanten α‑Oszillationen von okzipital/parietal zu den frontalen Regionen des Gehirns.

Während der Ausleitung einer Allgemeinanästhesie verschieben sich die dominanten δ‑Wellen von okzipital/parietal zu den frontalen Regionen des Gehirns.

Während der Ausleitung einer Allgemeinanästhesie verschieben sich die dominanten α‑Oszillationen von okzipital/parietal zu den frontalen Regionen des Gehirns.

Insbesondere bei einem Schmerzreiz verschieben sich die dominanten δ‑Wellen von okzipital/parietal zu den frontalen Regionen des Gehirns.

Welchen prädiktiven Wert hat das intraoperative EEG für den (postoperativen) Verlauf?

Retrospektiv besteht ein Zusammenhang zwischen der Dauer der intraoperativen EEG-Suppression und der Inzidenz eines postoperativen Delirs.

Längerfristige intraoperative EEG-Suppressionen erzeugen irreversible kognitive Defizite.

Das intraoperative EEG hat keinen prädiktiven Wert hinsichtlich des postoperativen Verlaufs.

Ein intraoperatives isoelektrisches EEG weist auf eine toxische Schädigung von Neuronen hin, die postoperativ irreversibel ist.

Ein intraoperatives Burst-Suppression-EEG weist häufig auf eine zerebrale Hypoperfusion hin.

Was kann typischerweise keine Artefakte im EEG auslösen?

Elektrokauterisation

Elektrische Aktivität der Herzmuskulatur

Konvektionswärmer

Berühren der EEG-Elektroden

Neuromuskuläre Blockade

Welchen Einfluss haben das Alter und die Komorbiditäten eines Patienten auf das intraoperative EEG?

Je älter der Patient, desto mehr Leistung hat man während einer Vollnarkose im δ‑Bereich.

Mit zunehmendem Alter nimmt die Spitzenfrequenz der in Allgemeinanästhesie zu erkennenden α‑Wellen zu.

Bei älteren Patienten nimmt die Zahl der „slow waves“ während der Allgemeinanästhesie zu.

Chronische Niereninsuffizienz hat typischerweise keinen Einfluss auf das intraoperative EEG.

Die Veränderung der α‑Spindel-Frequenz in Abhängigkeit von der Zielortkonzentration ist mit zunehmendem Alter oft schwieriger zu erkennen.

Welche Besonderheit trifft auf α‑Oszillationen/α-Spindeln zu?

Im Gegensatz zu klassischen Schlafspindeln sind α‑Oszillationen während einer Allgemeinanästhesie nur von kurzer Dauer.

Ein abrupter Verlust der frontalen α‑Oszillationen deutet auf eine unzureichende muskuläre Relaxierung hin.

Frontale α‑Oszillationen sind typischerweise während Narkosen mit Ketamin zu beobachten.

Die Frequenz der α‑Spindeln nimmt mit steigender Anästhetikakonzentration im Gehirn ab.

α‑Spindeln liegen per definitionem in einem Frequenzbereich von 1–4 Hz.
